# Hématome de la fosse cérébrale postérieur d'un nouveau-né après naissance par ventouse

**Published:** 2012-07-24

**Authors:** Faycal Moufid, Noureddine Oulali

**Affiliations:** 1 Département de Neurochirurgie, Faculté de Médecine Oujda, Maroc

**Keywords:** Ventouse, hématome sous-dural, fosse cérébrale postérieure, vacuum extraction, subdural hematoma, posterior cerebral fossa

## Abstract

L'hématome sous-dural est fréquent chez l'adulte, par ailleurs son incidence chez le nouveau-né est rare, environ une centaine de cas dans la littérature. Nous rapportons un cas d'hématome sous-dural de la fosse cérébrale postérieur chez un n-né de 1 mois après naissance par ventouse, traité par abord chirurgicale de la FCP par un seul trou de trépan élargie.

## Introduction

L'hématome sous-dural est fréquent chez l'adulte, par ailleurs son incidence chez le nouveau-né est rare, environ une centaine de cas dans la littérature. Nous rapportons un cas d'hématome sous-dural de la fosse cérébrale postérieur chez un n-né de 1 mois après naissance par ventouse, traité par abord chirurgicale de la FCP par un seul trou de trépan élargie.

## Patient et cas clinique

**Figure 1:**
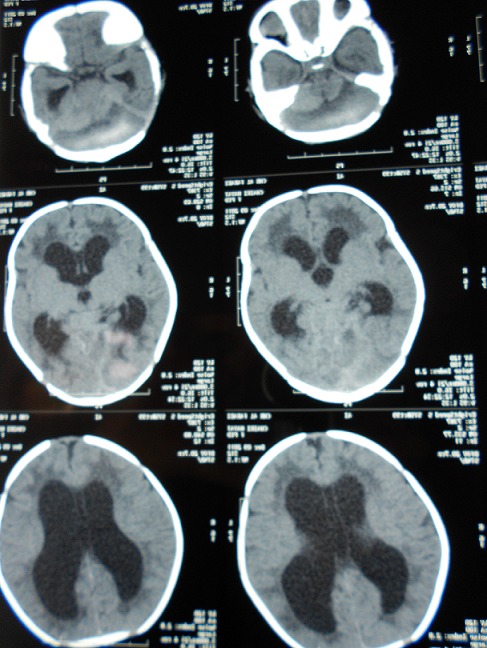
Scanner cerebral montrant un hematoma sous-dural de la fosse cérébrale postérieure avec hydrocéphalie triventriculaire

**Figure 2:**
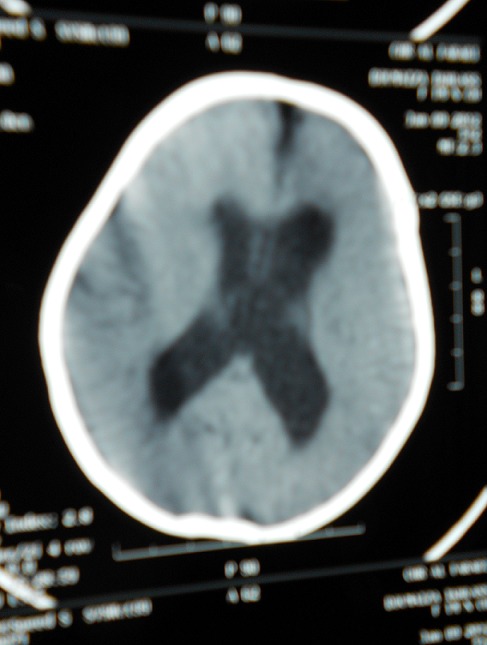
Scanner cerebral à j+2 montrant la reduction de l'hydrocéphalie

**Figure 3:**
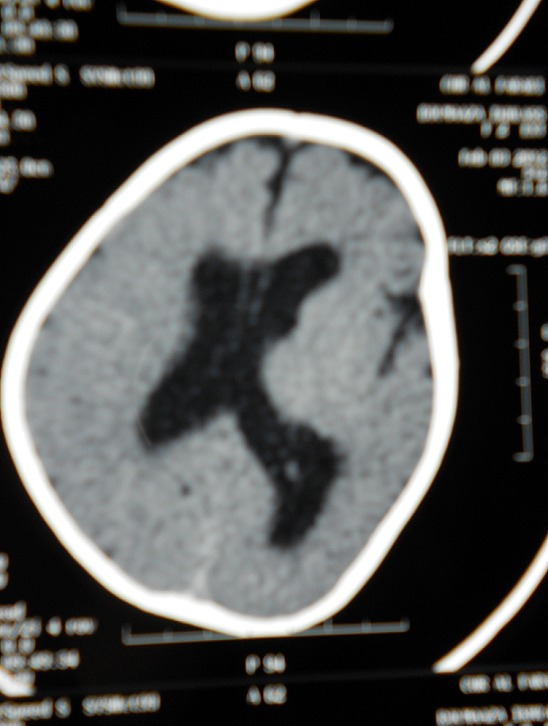
Scanner cerebral montrant la disparition de l'hydrocéphalie

Nourrisson de 1 mois, accouché par voie basse par ventouse, qui a présenté une macrocranie avec fontanelle antérieur bombante, une hypotonie généralisé et une parésie de la troisième paires crânienne dans un contexte d'apyrexie. Le bilan de la crase sanguine été normale. Le scanner cérébral a objectivé un hématome sous –durale chronique (HSDC) de la Fosse cérébrale postérieure (FCP) avec hydrocéphalie tri-ventriculaire active (Figure 1). Le patient a bénéficié d'une trépanation occipitale avec évacuation d'un hématome sous durale sous forme de sang lysé. Le malade en post-opératoire est devenu plus réactif, fontanelle moins bombante. Le scanner cérébral de contrôle à J+2 a montré l'évacuation de l'hématome et légère réduction de la dilatation ventriculaire (Figure 2). Le scanner cérébral à 1 mois a montré la disparition de l'hydrocéphalie (Figure 3).

## Discussion

Sur le plan physiopathologique, trois mécanismes ont été invoqués dans la formation de l'HSD de la FCP: le premier mécanisme est le traumatisme des veines anastomotiques du cervelet ou de la tente qui s'abouche dans le sinus dural [1, 2], le deuxième est la rupture ou la déchirure des grandes veines anastomotique telle le sinus droit ou la veine de Gallien [1, 2], le troisième a été décrit par Hemsath et al [3] qui évoque la compression de l'occiput contre la symphyse pubienne de la mère lors du passage de la tête du nouveau-né à travers la filière génitale. Dans la littérature il y a une centaine de cas d'HSD de la FCP car les nouveau-nés qui naissent par ventouse et qui n'ont pas de signes Clinique ne sont pas explores sur le plan radiologique. Parmi les séries les plus grandes, celle de Perrin-Richard [4] portant sur 15 cas dont 10 nouveau-nés par ventouse avec des signes de compression du tronc cérébrale (apnées, cyanose, bradycardie); dans d'autres cas, des causes de déficit en vitamine K, thrombocytopénie, dont le diagnostic a été porté tardivement, au 6e mois, par une macrocranie et une ataxie du tronc. Dans notre cas, aucun de ces facteurs n'a été noté. L'étude de Perrin-Richard [4] le diagnostic de l'HSD de la FCP a été fait dans tous les cas grâce au scanner cérébrale, aucun des cas n'a bénéficié d'une d'échographie trans-fontanellaire (ETF) comme ce fut le cas de notre patient. L'ETF reste malgré tout l'examen de choix [5] car elle n'est pas invasive, peut être effectué au lit du malade et ne nécessite pas de prémédication. Avrahami et al [6] ont réalisé un scanner cérébral dans les 12 à 24 premières heures chez dix nouveau-nés bien portant, nés à terme, par ventouse; une hémorragie de la FCP a été constatée chez tous ces nouveau-nés; le scanner cérébrale de contrôle réalisé une semaine plus tard montrait une résorption complète de l'hémorragie sans aucune autre atteinte cérébrale. Les examens cliniques et scannographiques après 1 an étaient normaux. Les auteurs concluent que ces hémorragies ont un pronostique favorable chez les enfants ne présentant aucun signe clinique. Dans la série de Ezzedine et al [5]; les nouveau-nés ayant un volumineux hématome et des signes cliniques de compression du tronc cérébrale ont bénéficié d'un acte chirurgicale d'évacuation sur la FCP. Govert et al et Taouka et al [2, 7, 8] proposent une chirurgie de la FCP au cas d'hématome supérieur à 1.5cm, hydrocéphalie obstructive avec syndrome d'hypertension intracrânienne. Ghani et al [9] ont rapporté un cas d'hématome sous dural de la FCP avec hydrocéphalie obstructive chez un nourrisson de 2 semaines traité initialement par un shunt ventriculaire mais compliqué d'un engagement rétrograde motivant une craniectomie en urgence pour évacuer l'hématome. Dans notre cas, l'évacuation chirurgicale de l'hématome a été indiquée, argumenté par hydrocéphalie obstructive avec syndrome d'hypertension intracrânienne. On a opté pour l'évacuation de l'hématome de la FCP et non la dérivation ventriculaire par crainte d'un engagement rétrograde. Le suivi de notre patient sur 6 mois retrouve un développement psychomoteur normal avec une discrète parésie de la troisième paire crânienne. L'évolution rapporté par Ezzedine et al [5] retrouve un développement psychomoteur normal pour 6 patients sur 7 avec un recul moyen de 22 mois, un patient a gardé une ataxie cérébelleuse avec strabisme. Notre patient nous a été adressé du service de pédiatrie, il serait intéressant de réaliser une étude prospective sur l'impact de la ventouse et ses conséquences hémorragique, surtout les hématomes de la FCP en réalisant de façon systématique une ETF chez les enfants nés par ventouse.

## Conclusion

Une ETF doit être systématique chez les enfants nés par ventouse avec ou en absence de signes Clinique de compression du tronc cérébrale. Ça serait le garent pour détecter les hémorragies cérébrale du n-né spécialement les hématomes de la FCP. Il est judicieux en cas d'indication chirurgicale d'évacuer l'hématome de la FCP et non la dérivation ventriculaire pour éviter l'engagement rétrograde.
